# National and Subnational Burden of Female Breast Cancer in Iran from 2010 to 2021

**DOI:** 10.3390/diseases14010015

**Published:** 2025-12-31

**Authors:** Zahra Pasokh, Afrooz Mazidimoradi, Mohsen Hamidian, Zahra Shahabinia, Mohaddeseh Kiani, Hamid Salehiniya

**Affiliations:** 1 Department of Epidemiology, Shiraz University of Medical Sciences, Shiraz 8433471946, Iran; zahr4.p98@gmail.com; 2 Department of Health Assistant, Shiraz University of Medical Sciences, Shiraz 8433471946, Iran; mazidimoradi.8836@gmail.com; 3 Clinical Research Development Unit, Amir Al Momineen Hospital, Zabol University of Medical Sciences, Zabol 9861615881, Iran; 4 Geriatric Health Research Center, Birjand University of Medical Sciences, Birjand 9717853577, Iran; 5 Birjand University of Medical Sciences, Birjand 9717853577, Iran; 6 Department of Epidemiology and Biostatistics, School of Health, Social Determinants of Health Research Center, Birjand University of Medical Sciences, Birjand 9717853577, Iran

**Keywords:** incidence, mortality, disease burden, breast cancer

## Abstract

*Background and Objectives*: Female breast cancer (FBC) is an increasing public health concern in Iran, with notable geographic disparities that necessitate comprehensive burden assessments at national and provincial levels. This study presented the national and subnational burden and changes in FBC burden from 2010 to 2021 in Iran in comparison with global data. *Materials and Methods*: The GBD (2021) data on female BC were extracted from the Global Health Data Exchange (GHDx) query tool. Age-standardized incidence, deaths, prevalence, and adjusted years of life with disabilities (DALYs) rates (per 100,000) of FBC were extracted. Data were extracted globally, by continents, for Iran and its provinces, from 2010 to 2021. *Results*: Although the global FBC burden indicators remained almost stable, in Iran, there was a nearly twofold rise in incidence and prevalence and notable rises in mortality and DALYs. This study showed significant variation at the provincial level; Tehran, Qom, and Alborz consistently had the highest incidence, prevalence, mortality, and DALY rates, whereas Sistan and Baluchistan, Chahar Mahaal and Bakhtiari, Kohgiluyeh and Boyer-Ahmad, and Zanjan had the lowest rates. During 2010–2021, the provinces of Golestan, Ardebil, Sistan and Baluchistan, West Azarbayejan, Kohgiluyeh and Boyer-Ahmad, and North Khorasan experienced the most increasing trend in BC burden, while Yazd and Semnan showed smaller increases or modest decreases. *Conclusions*: The rising FBC burden in Iran underscores the urgent need to strengthen cancer registries, expand screening programs, ensure equitable resource distribution, and implement targeted regional interventions focused on modifiable risk factors and early detection to reduce health disparities nationwide.

## 1. Introduction

Female breast cancer (FBC) remains a major global health challenge, with 2.3 million new cases and 685,000 deaths reported among women in 2020. Representing 25% of all female cancer diagnoses, it is the leading cancer in women worldwide. The burden is rising most markedly in developing settings, and FBC contributes more disability-adjusted life years (DALYs) in women than any other cancer type [1,2]. The burden of FBC has substantially increased from 1990–2021, with the greatest increases seen in regions with middle and low Socio-Demographic Index (SDI), likely resulting from disparities in access to prevention, diagnosis, and treatment services [3]. Nearly half (45.4%) of FBC cases are diagnosed in Asia [4]. In Asia, there is a positive correlation between the Human Development Index and FBC incidence rates, while countries with a low Human Development Index exhibit the highest mortality rates [5]. According to GLOBOCAN 2020, FBC ranked as the most prevalent malignancy in Iran, accounting for 12.9% of all incident cancers. It was the fifth leading cause of cancer mortality, responsible for 6.1% of cancer-related deaths, with age-standardized incidence and mortality rates of 35.8 and 10.8 per 100,000, respectively [6]. Iranian women are diagnosed with FBC about a decade earlier than their European counterparts, suggesting a disproportionately high disease burden in Iran [7]. Geographic clustering in Iran shows the highest age-standardized incidence rates (ASIRs) in central provinces such as Tehran and Alborz (~72 per 100,000), while southeastern provinces, including Khorasan and Sistan and Balochestan, have the lowest rates (5–11 per 100,000) [8,9,10].

A variety of factors, including genetic predisposition, environmental exposures, lifestyle choices, and inequalities in healthcare access, play major roles in FBC mortality and morbidity across regions and countries [11]. Recent health-related theories highlight that, beyond biological pathways, non-medical determinants also shape FBC risk. These determinants encompass issues such as social marginalization, psychological stress, insecure employment, job loss, the psychosocial work environment, social cohesion and support, substance use, transportation, urbanization, and the migration of minority groups [12,13]. An analysis of the Iran Health Insurance Organization database demonstrated rising trends in FBC incidence, prevalence, and mortality, resulting in substantial financial strain on households and increasing the risk of impoverishing healthcare expenditures [14].

Given the regional variation in FBC burden across Iran, it is essential to generate accurate national and subnational estimates to capture the current status of the disease. Such evidence is critical for identifying geographic disparities, guiding equitable resource allocation, and informing tailored strategies for prevention, early detection, and treatment. This study utilizes comprehensive data from the Global Burden of Disease study 2021 (https://ghdx.healthdata.org/gbd-2021, accessed on 17 July 2025) to analyze national and subnational trends in FBC incidence, prevalence, mortality, and disability-adjusted life years (DALYs) in Iran, providing insights to strengthen national cancer control policies and reduce inequities in outcomes.

## 2. Materials and Methods

This research utilized GBD 2021 data to examine FBC metrics, including incidence, mortality, and prevalence rates, along with disability-adjusted life years (DALYs), all expressed per 100,000 population for 2021, plus percentage shifts from 2010 to 2021 across Iranian provinces. BC statistics from GBD 2021 were retrieved via the GHDx query tool. The GBD 2021 framework offers systematic estimates for 286 death causes, 369 disease and injury types, and 87 risk factors across 204 countries and territories. Further details on data inputs are available through the GBD 2021 sources page (http://ghdx.healthdata.org/gbd-2021/data-input-sources) on 17 July 2025 [15,16]. GBD 2021 defines FBC using ICD-10 codes C50.0–C50.9 [17]. Estimates draw from cancer registries, vital registration, and other sources; in Iran, data primarily come from the national pathology-based registry, with variable provincial coverage and potential underreporting addressed via modeling [18]. Mortality is estimated using the Cause of Death Ensemble model (CODEm), incidence via mortality-to-incidence ratios, and prevalence/DALYs through DisMod-MR Bayesian meta-regression. Uncertainty intervals (95% UIs) are derived from 1000 posterior draws, reflecting data and model variability [17]. In GBD, DALYs represent a standardized measure akin to quality-adjusted life years, quantifying healthy years lost. One DALY equals one year of full health forfeited due to early mortality or disability of defined intensity and length. “Premature” mortality refers to deaths before the age expected in a reference population with the global highest life expectancy at birth, currently Japan. Total DALYs for a condition in a population are calculated by summing years of life lost (YLLs) from premature deaths and years lived with disability (YLDs) of established severity and duration [19,20]. For each classification, indicators were presented separately. The incidence, death, and DALY rates of BC were reported per 100,000 individuals. To ensure comparability and eliminate the effect of age distribution, ASRs were employed. For each classification, indicators were presented separately. We used the relative changes (%) between years to show comparative changes in age-standardized rates. The relative changes were calculated by dividing the value of the absolute difference by the value of the year of origin, which was multiplied by 100 [21]. Data are reported in values and a 95% confidence interval (CI).

The research received approval from the ethics committee at Birjand University of Medical Sciences (code: IR.BUMS.REC.1400.316). Since the analysis involved de-identified routine electronic records, individual patient consent was waived.

## 3. Results

### 3.1. Female Breast Cancer (FBC) Incidence in Iran and Globally

As shown in Table 1, global FBC incidence in 2021 reached approximately 2.1 million cases, with an age-standardized incidence rate (ASIR) of 46.4 per 100,000 women. This represented a slight increase from about 1.6 million cases and an ASIR of 45.3 in 2010, indicating relative stability in global age-standardized rates.

Asia contributed roughly 934,000 new cases in 2021 (approximately 45% of the global total), marking a 1.6-fold rise since 2010. Asia’s ASIR increased from 29.1 to 35.2 per 100,000 (a 21% rise). Despite this growth, Europe and the Americas maintained higher ASIRs.

In Iran, incidence nearly doubled, rising from around 12,000 cases in 2010 to about 25,000 in 2021. The ASIR increased substantially from 36.2 to 52.9 per 100,000 (a 46% overall rise over the period), surpassing global trends. However, a 7% decline in ASIR was observed between 2019 and 2021.

Subnationally, with regard to the highest burden, Tehran province had the highest crude incidence (over 7000 cases) and rate (85.4 per 100,000) in 2021, with a 43% ASIR increase since 2010. Qom and Alborz followed closely, with rates of approximately 66 and 63 per 100,000, respectively. The lowest ASIRs were recorded in Sistan and Baluchistan (21 per 100,000), Chahar Mahaal and Bakhtiari (22 per 100,000), Zanjan (26 per 100,000), and Kohgiluyeh and Boyer-Ahmad (27 per 100,000). The largest relative ASIR rises occurred in West Azarbaijan and Golestan (both 66%), Kohgiluyeh and Boyer-Ahmad (64%), Ardebil (64%), Sistan and Baluchistan (63%), and North Khorasan (59%). In contrast, Yazd and Semnan exhibited the smallest increases (around 25%). Notably, all provinces showed declining ASIR trends from 2019 to 2021.

### 3.2. FBC Death in Iran and Globally

According to the findings in Table 2, global FBC deaths rose from approximately 511,000 in 2010 to 661,000 in 2021, while age-standardized mortality rates remained relatively stable at around 15 per 100,000. Asia accounted for the largest share of these deaths, increasing from about 211,000 to 306,000, accompanied by a modest 7% rise in the mortality rate. In contrast, Africa, Europe, and the Americas generally exhibited higher mortality rates, although Europe and the Americas showed declining trends from 2010 to 2019.

In Iran, the number of deaths increased from around 2400 in 2010 to 4000 in 2021, with the age-standardized mortality rate rising 15% (from 8.2 to 9.4 per 100,000). However, an 8% decline was observed from 2019 to 2021. In 2021, Tehran recorded the highest mortality both in absolute numbers (around 1060 deaths) and age-standardized rate (13.6 per 100,000), reflecting a 15% increase since 2010. Qom and Alborz followed with the highest age-standardized death rates (approximately 12.5 and 11.2 per 100,000, respectively). The lowest rates were seen in Chahar Mahaal and Bakhtiari (3.3 per 100,000), Kohgiluyeh and Boyer-Ahmad (4.2), Zanjan (4.7), and Sistan and Baluchistan (5.6).

From 2010 to 2019, the largest relative increases in mortality occurred in Golestan (36%), Ardebil (33%), Sistan and Baluchistan (30%), West Azarbayejan (28%), and North Khorasan (27%), while Yazd, Qom, and Semnan experienced slight decreases (2–4%). Notably, all provinces recorded mortality declines from 2019 to 2021.

### 3.3. FBC Prevalence in Iran and Globally

According to Table 3, the global number of prevalent FBC cases rose from approximately 15 million in 2010 to 20 million in 2021, with the age-standardized prevalence rate (ASPR) increasing slightly from 440 to 451 per 100,000 women. Asia accounted for the largest share, with around 9 million prevalent cases in 2021 (43% of the global total)—a substantial rise since 2010—and its ASPR increased by 22%, from 270 to 330 per 100,000 women. In contrast, Europe and the Americas had much higher ASPRs, at 723 and 694 per 100,000 women, respectively.

In Iran, the number of prevalent cases nearly doubled over the study period, and the national ASPR rose by 46%, from 352 to 513 per 100,000 women, although a modest 5% decline in ASPR was observed from 2019 to 2021. Subnational variation was considerable: Tehran had the highest prevalence and ASPR (approximately 825 per 100,000), followed by Qom and Alborz (with ASPRs around 630 and 613 per 100,000, respectively), while the lowest ASPRs were recorded in Sistan and Baluchistan (204 per 100,000), Chahar Mahaal and Bakhtiari, Zanjan, and Kohgiluyeh and Boyer-Ahmad.

The fastest ASPR increases occurred in Sistan and Baluchistan, Kohgiluyeh and Boyer-Ahmad, Golestan, West Azarbayejan, and North Khorasan (61–66%), whereas Semnan and Yazd showed the smallest rises (28–30%). Consistent with incidence trends, all provinces experienced a declining ASPR from 2019 to 2021.

### 3.4. FBC DALYs in Iran and Globally

As observed in Table 4, global female breast cancer (FBC) disability-adjusted life years (DALYs) increased from 15.9 million in 2010 to 20.3 million in 2021, while the age-standardized DALY rate per 100,000 population remained relatively stable at approximately 456. In Asia, the age-standardized DALY rate rose from around 361 to 391 per 100,000 (an 8% increase), and Africa recorded the largest rise at 13%. In contrast, Europe and the Americas showed decreases of 14% and 6%, respectively.

In Iran, DALYs increased substantially from approximately 92,000 in 2010 to 153,000 in 2021, with the age-standardized DALY rate rising from 281 to 327 per 100,000 (a 17% overall increase). However, a temporary 8% reduction was observed between 2019 and 2021.

Tehran consistently had the highest DALY burden, reaching around 38,000 DALYs and an age-standardized rate of 450 per 100,000 in 2021 (a 16% increase since 2010). Qom and Khuzestan also ranked among the highest, with rates of approximately 408 and 370 per 100,000, respectively.

The lowest age-standardized DALY rates were observed in Chahar Mahaal and Bakhtiari (126 per 100,000), Kohgiluyeh and Boyer-Ahmad (162), Zanjan (168), and Sistan and Baluchistan (208).

Provinces with the steepest upward trends in age-standardized DALY rates included Golestan (39% increase), Ardebil (36%), Sistan and Baluchistan (33%), West Azarbayejan (30%), and North Khorasan (30%). Yazd and Semnan showed minor decreases (3% and 1%, respectively). Notably, declines in DALY rates were observed across all provinces between 2019 and 2021.

## 4. Discussion

Between 2010 and 2021, global FBC burden indicators showed modest but meaningful trends. Incidence cases increased slightly, while age-standardized incidence rates per 100,000 remained largely stable. Similarly, prevalence numbers rose, but prevalence rates increased only marginally. These patterns suggest that the rise in incidence is primarily driven by population growth and aging, whereas improvements in early detection and treatment have enhanced survival rates, contributing to higher prevalence [22].

Although absolute mortality numbers increased, age-standardized mortality rates declined slightly, reflecting advances in detection and management. Disability-adjusted life years (DALYs) remained stable, as gains in outcomes offset the higher incidence. These findings align with those of Ghoncheh et al., who noted that rising FBC incidence does not necessarily translate to higher mortality, particularly in countries with high Human Development Index (HDI) levels, where incidence is greater but mortality tends to be lower compared to less developed regions [23].

Regionally, age-standardized incidence and prevalence rates (ASIR and ASPR) were highest in Europe and the Americas, whereas Africa recorded the highest age-standardized mortality and DALY rates. From 2010 to 2021, Europe and the Americas generally exhibited downward trends in incidence, prevalence, mortality, and DALY rates, in contrast to sharp increases observed in Asia and Africa. These regional disparities are consistent with global analyses reporting sustained rises in FBC burden, especially in low- and middle-income countries (LMICs) [4,5].

This contrast is partly explained by differences in early detection programs, which are far less prevalent in LMICs than in high-income countries (HICs) [24]. As a result, advanced-stage diagnoses are more common in LMICs, where fewer than half of cases are detected at stages I or II, compared to approximately 70% in HICs [25].

In Iran, the burden of FBC showed markedly different trends. We observed nearly a twofold increase in incidence and prevalence, alongside substantial rises in mortality and DALYs. These findings are consistent with prior national estimates, including data from the Global Burden of Disease Study 2019, which documented marked increases in age-standardized incidence, mortality, and DALY rates from 1990 to 2019 [26], as well as observational studies from 2009 to 2019, reporting consistent upward trends across multiple burden metrics [27].

Subnationally, considerable variation existed across Iranian provinces between 2010 and 2021. Provinces such as Tehran, Qom, and Alborz consistently exhibited the highest incidence, prevalence, mortality, and DALY rates, with notable increases over time. Higher socioeconomic status and better healthcare infrastructure in these areas likely facilitate greater case detection (contributing to elevated incidence and prevalence), yet paradoxically do not always translate to lower mortality or DALYs, underscoring the need to examine treatment quality and access to advanced care. In addition, the impact of risk factors such as urbanization, fertility patterns, obesity, and hormone use should be explored [28,29,30].

These elevated rates in high-socioeconomic development index (SDI) regions may also reflect greater exposure to urbanization-related risk factors—such as higher fat consumption, smoking, low physical activity, environmental pollutants, later childbearing, reduced breastfeeding, obesity, and hormone therapy—as well as increased awareness and screening uptake [26,31,32,33,34]. Supporting this, Khorrami et al. found that provinces with higher urban livability, including Tehran, Mazandaran, and East Azerbaijan, had correspondingly higher FBC incidence rates [35]. Such disparities highlight the importance of region-tailored public health strategies: enhancing screening infrastructure in underserved areas while promoting healthy lifestyles in urbanized, high-risk regions.

In contrast, provinces with the lowest rates—such as Sistan and Baluchistan, Chahar Mahaal and Bakhtiari, Kohgiluyeh and Boyer-Ahmad, and Zanjan—often feature different demographic and lifestyle profiles or face barriers to screening and diagnosis, potentially leading to underreporting. Meanwhile, certain provinces—including Golestan, Ardebil, Sistan and Baluchistan, West Azarbayejan, Kohgiluyeh and Boyer-Ahmad, and North Khorasan—emerged as hotspots with rapidly escalating incidence, prevalence, mortality, and DALY rates. This surge can be attributed in part to the expansion of national and regional FBC screening programs starting around 2013–2015, which initially emphasized clinical breast examination and mammography for women over 40 and have gradually reached less developed areas [10,36,37,38]. Improved diagnostic access has enhanced case detection and reporting—for example, in Sistan and Baluchistan, recorded incidence rose from 98 cases in 2010 to 234 in 2021. Lifestyle transitions, population aging, and growing awareness likely also contribute to genuine increases. Thus, these trends reflect both improved surveillance and a true rising burden, necessitating targeted regional interventions. Provinces such as Yazd and Semnan, which showed smaller increases or modest declines, may benefit from effective local interventions or distinct risk profiles; these patterns merit further study.

Finally, the COVID-19 pandemic disrupted FBC care worldwide, reducing screening, diagnoses, surgeries, chemotherapy, and radiotherapy [39,40]. Apparent declines in burden indicators in Iran from 2019 to 2021 are likely attributable to these disruptions in screening, treatment, and reporting rather than genuine improvements in outcomes [41]. Despite these temporary effects, the overall upward trajectory of FBC burden in Iran remains a critical public health concern requiring urgent, focused action.

It is notable that today, biomedical advances have transformed FBC outcomes through improved screening, precision diagnostics, targeted therapies, and prevention strategies. Early detection benefits from 3D mammography [42], AI-enhanced risk prediction [43], and liquid biopsies detecting circulating tumor DNA [44,45], enabling personalized screening and reducing overtreatment of low-risk DCIS. Treatment has shifted from broad chemotherapy to highly targeted approaches: oral SERDs (Elacestrant, Imlunestrant), CDK4/6 inhibitors (Ribociclib, Abemaciclib), PI3K inhibitors (Inavolisib), and antibody-drug conjugates (Trastuzumab Deruxtecan, Datopotamab Deruxtecan) significantly extend progression-free and overall survival in HR-positive, HER2-low, and triple-negative subtypes [46]. Immunotherapy with pembrolizumab improves survival in PD-L1-positive cases, while emerging CAR-T and vaccine trials offer future promise. Risk-reducing agents (tamoxifen, anastrozole) and diverse genetic studies further support prevention in high-risk populations [47]. Collectively, these innovations have increased five-year survival to >90% for localized disease [48] and reduced mortality by ~40% since the 1990s [49], marking a shift toward personalized, less toxic, and more effective FBC management. On the other hand, social determinants of health (SDOH) that drive disparities in diagnosis and outcomes play a pivotal role in addressing FBC in Iran. Sociocultural norms, psychological stressors, and socioeconomic disparities profoundly influence screening, treatment adherence, and survivorship. Qualitative studies reveal that cultural taboos around sexuality and body image exacerbate psychological distress among survivors, hindering holistic care [50]. Risk perceptions shaped by social determinants—such as family history, stress, and environmental factors—often lead to delayed diagnosis, with metaphysical beliefs compounding barriers in low-resource settings [51]. Psychosocial mediators like self-efficacy and social support mitigate disparities in post-diagnosis health behaviors, promoting resilience via health belief and stress-coping models [52]. Recent geospatial analyses underscore how education, income, and pollution intersect to drive regional incidence variations, urging equity-focused policies [53].

The principal strength of this study lies in its comprehensive analysis of FBC burden in Iran using the GBD 2021 dataset, which provides robust, nationally and provincially disaggregated data over 11 years. This allows for a nuanced understanding of both temporal trends and regional disparities across multiple burden metrics, including incidence, prevalence, mortality, and DALYs. Such detailed subnational insights are critical for informing targeted public health strategies and improving resource allocation. Nonetheless, the study faces inherent limitations related to data quality and completeness, particularly variability across provincial cancer registries, which may lead to underreporting or misclassification. The potential impact of reporting delays and healthcare disruptions, including those caused by the COVID-19 pandemic, could affect temporal trend accuracy. Additionally, reliance on modeled estimates like DALYs might not fully capture local variations in disease outcomes. The absence of detailed socioeconomic and behavioral data in this study limits the ability to fully explain the underlying causes of regional disparities in FBC burden. Socioeconomic status, access to healthcare, screening practices, and lifestyle factors are known to significantly influence FBC incidence and outcomes in Iran. Moreover, the ecological, aggregated nature of the data restricts causal interpretations, emphasizing the need for future research employing individual-level, longitudinal designs that incorporate these critical determinants to better understand and address disparities in FBC epidemiology.

## 5. Conclusions

Given the escalating FBC burden in Iran, alongside persistent provincial disparities in incidence (ranging widely across regions) and socioeconomic inequalities affecting access to care, it is imperative to prioritize enhanced subnational surveillance and resource allocation tailored to regional needs. Key modifiable risk factors underscore the need for targeted interventions addressing metabolic and lifestyle influences. Strengthening population-based cancer registries, expanding equitable screening coverage (particularly in underserved low-SDI provinces), improving timely treatment access, and boosting public awareness campaigns are essential. Focusing on early detection strategies and modifiable risks will be critical to curbing nationwide disease growth. Ultimately, implementing region-specific, evidence-based policies and promoting equitable healthcare delivery can mitigate disparities, enhance survival rates, and improve outcomes across Iran.

## Figures and Tables

**Table 1:** Comparison of incidence cases and rates per 100,000 of female breast cancer based on provinces in 2010 and 2021.

Location	2010		2021		% Changes	
	Incidence Number (95%UI)	Incidence Rate per 100,000 (95%UI)	Incidence Number (95%UI)	Incidence Rate per 100,000 (95%UI)	2010–2021 (95%UI)	2019–2021 (95%UI)
Global	1,570,648	45.31	2,082,737	46.40	0.02	0.00
	(1,498,484–1,623,142)	(43.17–46.85)	(1,940,351–2,225,083)	(43.26–49.56)	(−0.04–0.09)	(−0.06–0.07)
**Continents**						
Africa	78,193	28.02	140,210	34.71	0.24	0.04
	(71,139–86,160)	(25.68–30.86)	(121,834–160,211)	(30.58–39.22)	(0.11–0.4)	(−0.05–0.13)
America	398,504	74.43	478,792	69.80	−0.06	0.00
	(376,358–410,581)	(70.56–76.59)	(444,691–502,368)	(65.38–73.15)	(−0.09–−0.03)	(−0.02–0.03)
Asia	586,140	29.13	934,358	35.23	0.21	0.02
	(553,706–622,442)	(27.47–30.93)	(837,387–1,050,748)	(31.62–39.59)	(0.07–0.37)	(−0.11–0.19)
Europe	503,939	75.04	524,971	69.51	−0.07	−0.04
	(477,047–519,320)	(71.9–76.99)	(484,317–552,172)	(65.67–72.78)	(−0.11–−0.04)	(−0.06–−0.01)
Iran (Islamic Republic of)	11,848	36.20	24,635	52.87	0.46	−0.07
	(10,869–12,839)	(33.09–39.16)	(22,074–27,581)	(47.34–59.18)	(0.29–0.65)	(−0.16–0.05)
**Iranian Provinces**						
Alborz	458	46.00	1048	63.49	0.38	−0.06
	(383–552)	(38.36–55.08)	(767–1395)	(47.24–84.01)	(0–0.92)	(−0.3–0.28)
Ardebil	107	19.20	235	31.52	0.64	−0.06
	(89–127)	(16.17–22.76)	(183–288)	(24.72–38.48)	(0.22–1.14)	(−0.23–0.17)
Bushehr	137	36.05	303	50.89	0.41	−0.06
	(114–163)	(30.52–42.36)	(237–391)	(40.15–64.74)	(0.08–0.82)	(−0.25–0.16)
Chahar Mahaal and Bakhtiari	58	15.93	121	22.35	0.40	−0.05
	(48–71)	(13.06–19.42)	(89–157)	(16.46–28.99)	(0.01–0.89)	(−0.3–0.21)
East Azarbayejan	545	30.44	1156	48.14	0.58	−0.07
	(446–643)	(25.02–35.93)	(869–1535)	(36.42–62.93)	(0.14–1.13)	(−0.3–0.2)
Fars	748	36.70	1503	51.52	0.40	−0.08
	(630–875)	(30.84–43.1)	(1143–1948)	(39.4–66.18)	(0.05–0.85)	(−0.31–0.24)
Gilan	569	41.53	1008	57.10	0.37	−0.08
	(466–683)	(33.97–49.28)	(780–1310)	(44.35–74.28)	(0.02–0.88)	(−0.31–0.25)
Golestan	192	25.15	451	41.84	0.66	−0.07
	(156–227)	(20.48–29.7)	(347–579)	(32.56–53.33)	(0.22–1.3)	(−0.26–0.15)
Hamadan	203	24.54	358	34.68	0.41	−0.06
	(175–238)	(21.21–28.8)	(280–456)	(27.12–43.86)	(0.07–0.8)	(−0.26–0.2)
Hormozgan	125	23.25	301	34.39	0.48	−0.05
	(97–157)	(18.14–29.13)	(213–409)	(24.67–46.25)	(−0.01–1.1)	(−0.26–0.26)
Ilam	54	23.38	116	33.52	0.43	−0.07
	(45–65)	(19.48–28.07)	(87–148)	(25.3–43.02)	(0.06–0.87)	(−0.28–0.18)
Isfahan	865	37.85	1667	52.49	0.39	−0.07
	(711–1016)	(31.23–44.31)	(1274–2207)	(40.38–69.22)	(0.02–0.83)	(−0.3–0.21)
Kerman	305	27.17	622	37.25	0.37	−0.06
	(255–364)	(22.95–32.09)	(467–800)	(28.38–47.57)	(0–0.81)	(−0.28–0.25)
Kermanshah	261	29.21	485	40.45	0.38	−0.06
	(225–305)	(25.25–33.95)	(372–617)	(31.19–51.33)	(0.03–0.82)	(−0.29–0.24)
Khorasan-e-Razavi	632	24.67	1362	38.49	0.56	−0.07
	(527–735)	(20.66–28.6)	(1041–1786)	(29.35–50.58)	(0.15–1.04)	(−0.28–0.2)
Khuzestan	605	34.81	1322	53.92	0.55	−0.06
	(510–711)	(28.91–40.84)	(1007–1715)	(41.16–69.36)	(0.1–1.07)	(−0.25–0.2)
Kohgiluyeh and Boyer-Ahmad	46	16.31	103	26.68	0.64	−0.05
	(37–56)	(12.96–20.06)	(76–134)	(19.76–34.63)	(0.16–1.3)	(−0.26–0.25)
Kurdistan	138	21.31	300	31.44	0.48	−0.06
	(116–161)	(18.08–24.9)	(231–384)	(24.06–40.1)	(0.1–0.95)	(−0.24–0.18)
Lorestan	175	24.22	363	36.12	0.49	−0.07
	(142–214)	(19.94–29.37)	(275–489)	(27.42–47.95)	(0.07–1.01)	(−0.3–0.2)
Markazi	202	30.14	360	41.23	0.37	−0.07
	(169–238)	(25.14–35.44)	(281–453)	(32.13–51.43)	(0.03–0.79)	(−0.29–0.17)
Mazandaran	687	43.46	1401	62.31	0.43	−0.08
	(579–794)	(36.67–49.99)	(1077–1784)	(48–79.12)	(0.08–0.87)	(−0.3–0.21)
North Khorasan	78	21.64	157	34.49	0.59	−0.05
	(65–94)	(18.06–26.21)	(117–204)	(25.83–44.78)	(0.14–1.14)	(−0.26–0.21)
Qazvin	169	32.31	355	46.49	0.44	−0.07
	(145–198)	(27.5–37.85)	(279–443)	(36.91–58.82)	(0.1–0.88)	(−0.27–0.21)
Qom	215	49.23	457	65.65	0.33	−0.04
	(179–254)	(41.29–57.52)	(350–590)	(51.1–84.36)	(0–0.74)	(−0.24–0.21)
Semnan	116	39.63	210	49.35	0.25	−0.14
	(98–137)	(33.58–46.52)	(155–273)	(36.14–64.62)	(−0.08–0.68)	(−0.38–0.15)
Sistan and Baluchistan	98	12.91	234	21.05	0.63	−0.02
	(79–123)	(10.35–15.89)	(170–310)	(15.37–27.35)	(0.13–1.3)	(−0.28–0.29)
South Khorasan	69	24.09	163	36.70	0.52	−0.04
	(57–81)	(20.35–28.47)	(124–209)	(27.97–46.95)	(0.15–0.97)	(−0.24–0.2)
Tehran	3401	59.84	7265	85.37	0.43	−0.06
	(2773–4156)	(48.71–73.26)	(5592–9491)	(65.87–111.13)	(0.02–0.97)	(−0.31–0.29)
West Azarbayejan	304	23.15	701	38.33	0.66	−0.06
	(251–369)	(19.25–27.89)	(533–900)	(29.51–49.05)	(0.2–1.23)	(−0.26–0.22)
Yazd	206	46.50	344	58.28	0.25	−0.10
	(172–243)	(38.76–54.94)	(261–443)	(44.58–74.73)	(−0.06–0.69)	(−0.32–0.23)
Zanjan	82	18.21	164	26.14	0.43	−0.06
	(67–99)	(15.08–21.87)	(128–209)	(20.68–33.27)	(0.09–0.9)	(−0.24–0.18)

**Table 2:** Comparison of death cases and rates per 100,000 of FBC based on provinces in 2010 and 2021.

Location	2010		2021		% Changes	
	Death Number	Death Rate per 100,000	Death Number	Death Rate per 100,000	2010–2021	2019–2021
	(95%UI)	(95%UI)	(95%UI)	(95%UI)	(95%UI)	(95%UI)
Global	510,842	14.87	660,925	14.55	−0.02	−0.01
	(481,220–530,256)	(13.99–15.45)	(609,171–707,182)	(13.45–15.56)	(−0.07–0.03)	(−0.06–0.05)
**Continents**						
Africa	43,880	17.30	71,514	19.58	0.13	0.02
	(39,874–48,662)	(15.81–19.06)	(62,639–81,242)	(17.37–22.03)	(0.02–0.28)	(−0.07–0.11)
America	99,727	18.13	121,070	16.90	−0.07	−0.01
	(92,664–103,621)	(16.95–18.78)	(110,562–128,204)	(15.53–17.85)	(−0.1–−0.04)	(−0.03–0.02)
Asia	210,821	10.83	306,495	11.59	0.07	0.00
	(199,831–222,500)	(10.24–11.44)	(276,354–340,677)	(10.47–12.88)	(−0.03–0.19)	(−0.1–0.12)
Europe	155,132	20.64	160,388	18.13	−0.12	−0.03
	(143,571–161,651)	(19.42–21.34)	(143,199–170,875)	(16.61–19.23)	(−0.16–−0.09)	(−0.06–0.01)
Iran (Islamic Republic of)	2406	8.19	4017	9.42	0.15	−0.08
	(2244–2565)	(7.54–8.76)	(3623–4444)	(8.47–10.43)	(0.04–0.29)	(−0.16–0.01)
**Iranian Provinces**						
Alborz	81	10.07	155	11.19	0.11	−0.08
	(68–96)	(8.42–12.01)	(117–203)	(8.52–14.4)	(−0.18–0.52)	(−0.3–0.22)
Ardebil	22	4.34	40	5.79	0.33	−0.07
	(19–26)	(3.72–5.02)	(32–49)	(4.66–7.06)	(0.03–0.7)	(−0.23–0.14)
Bushehr	29	8.57	48	9.06	0.06	−0.08
	(25–33)	(7.36–9.86)	(38–60)	(7.25–11.37)	(−0.16–0.36)	(−0.25–0.14)
Chahar Mahaal and Bakhtiari	11	3.28	17	3.34	0.02	−0.07
	(9–13)	(2.72–3.91)	(13–22)	(2.53–4.31)	(−0.25–0.36)	(−0.3–0.21)
East Azarbayejan	126	7.97	209	9.56	0.20	−0.09
	(106–145)	(6.78–9.11)	(161–270)	(7.5–12.21)	(−0.1–0.58)	(−0.3–0.17)
Fars	158	8.74	250	9.43	0.08	−0.09
	(134–181)	(7.47–10.12)	(195–316)	(7.36–11.95)	(−0.17–0.39)	(−0.3–0.21)
Gilan	111	8.75	163	9.65	0.10	−0.09
	(93–129)	(7.4–10.15)	(127–210)	(7.56–12.32)	(−0.16–0.44)	(−0.3–0.19)
Golestan	45	6.53	88	8.85	0.36	−0.09
	(37–52)	(5.38–7.66)	(69–112)	(7–11.19)	(0.02–0.86)	(−0.26–0.12)
Hamadan	47	6.10	68	6.93	0.14	−0.07
	(41–54)	(5.29–6.99)	(54–84)	(5.5–8.52)	(−0.12–0.41)	(−0.26–0.17)
Hormozgan	30	6.34	53	6.94	0.09	−0.07
	(24–37)	(5.08–7.67)	(38–70)	(5.13–9.01)	(−0.24–0.49)	(−0.27–0.21)
Ilam	11	5.24	19	6.00	0.14	−0.08
	(9–13)	(4.41–6.13)	(14–23)	(4.62–7.53)	(−0.12–0.48)	(−0.29–0.16)
Isfahan	168	8.15	269	9.23	0.13	−0.08
	(140–193)	(6.82–9.32)	(206–346)	(7.11–11.69)	(−0.15–0.45)	(−0.29–0.18)
Kerman	68	7.03	115	7.74	0.10	−0.07
	(58–81)	(6.07–8.24)	(90–146)	(6.1–9.71)	(−0.16–0.41)	(−0.28–0.21)
Kermanshah	60	7.44	88	7.90	0.06	−0.08
	(52–68)	(6.51–8.5)	(71–113)	(6.31–10.1)	(−0.19–0.37)	(−0.29–0.23)
Khorasan-e-Razavi	159	6.87	258	8.00	0.17	−0.09
	(135–183)	(5.88–7.95)	(200–333)	(6.24–10.39)	(−0.14–0.52)	(−0.29–0.16)
Khuzestan	132	8.63	226	10.26	0.19	−0.08
	(113–152)	(7.34–9.9)	(174–289)	(7.96–12.85)	(−0.12–0.56)	(−0.25–0.15)
Kohgiluyeh and Boyer-Ahmad	8	3.35	14	4.23	0.26	−0.07
	(7–10)	(2.74–4.1)	(11–18)	(3.23–5.41)	(−0.11–0.72)	(−0.27–0.21)
Kurdistan	32	5.48	53	5.98	0.09	−0.07
	(28–37)	(4.73–6.31)	(42–66)	(4.75–7.44)	(−0.15–0.38)	(−0.25–0.14)
Lorestan	36	5.52	56	6.02	0.09	−0.08
	(30–42)	(4.7–6.44)	(43–72)	(4.65–7.74)	(−0.19–0.43)	(−0.31–0.19)
Markazi	45	7.21	64	7.59	0.05	−0.08
	(39–52)	(6.18–8.34)	(51–79)	(6.04–9.42)	(−0.17–0.36)	(−0.28–0.15)
Mazandaran	123	8.48	210	9.85	0.16	−0.08
	(106–140)	(7.28–9.62)	(167–264)	(7.84–12.26)	(−0.09–0.49)	(−0.29–0.17)
North Khorasan	19	5.83	31	7.38	0.27	−0.07
	(16–23)	(4.9–6.96)	(23–40)	(5.6–9.68)	(−0.09–0.68)	(−0.27–0.17)
Qazvin	36	7.69	59	8.40	0.09	−0.09
	(32–42)	(6.63–8.94)	(47–73)	(6.66–10.6)	(−0.13–0.4)	(−0.28–0.18)
Qom	47	12.93	75	12.52	−0.03	−0.06
	(41–54)	(11.28–14.64)	(59–95)	(10.12–15.82)	(−0.24–0.23)	(−0.23–0.16)
Semnan	25	9.18	36	8.98	−0.02	−0.15
	(21–29)	(7.87–10.66)	(26–46)	(6.53–11.52)	(−0.26–0.28)	(−0.37–0.14)
Sistan and Baluchistan	29	4.27	55	5.57	0.30	−0.06
	(23–35)	(3.48–5.14)	(41–72)	(4.21–7.24)	(−0.06–0.8)	(−0.29–0.24)
South Khorasan	17	6.27	31	7.27	0.16	−0.06
	(15–20)	(5.33–7.34)	(24–39)	(5.74–9.17)	(−0.13–0.46)	(−0.24–0.15)
Tehran	598	11.80	1059	13.55	0.15	−0.07
	(498–724)	(9.8–14.36)	(825–1352)	(10.57–17.29)	(−0.17–0.56)	(−0.3–0.27)
West Azarbayejan	69	6.08	127	7.77	0.28	−0.08
	(58–81)	(5.18–7.08)	(98–161)	(6.08–9.83)	(−0.03–0.69)	(−0.26–0.17)
Yazd	46	11.17	57	10.69	−0.04	−0.11
	(39–52)	(9.5–12.79)	(44–73)	(8.37–13.7)	(−0.25–0.24)	(−0.32–0.19)
Zanjan	18	4.25	27	4.65	0.09	−0.07
	(15–21)	(3.61–4.94)	(22–34)	(3.74–5.87)	(−0.17–0.44)	(−0.25–0.14)

**Table 3:** Comparison of prevalence cases and rates per 100,000 of FBC based on provinces in 2010 and 2021.

Location	2010		2021		% Changes	
	Prevalence Number	Prevalence Rate per 100,000	Prevalence Number	Prevalence Rate per 100,000	2010–2021	2019–2021
	(95%UI)	(95%UI)	(95%UI)	(95%UI)	(95%UI)	(95%UI)
Global	15,192,406	439.71	20,323,179	450.64	0.02	0.00
	(14,621,665–15,742,391)	(422.79–455.65)	(19,248,044–21,451,412)	(427.02–475.96)	(−0.02–0.08)	(−0.05–0.06)
**Continents**						
Africa	592,064	207.35	1,092,040	264.60	0.28	0.04
	(545,265–644,815)	(192.25–224.5)	(963,001–1,231,501)	(236.67–294.46)	(0.16–0.42)	(−0.03–0.12)
America	3,997,652	743.91	4,825,585	694.29	−0.07	0.00
	(3,828,620–4,131,727)	(714.12–768.5)	(4,579,846–5,062,610)	(660.9–726.24)	(−0.09–−0.04)	(−0.02–0.02)
Asia	5,397,585	270.34	8,765,541	330.01	0.22	0.02
	(5,132,530–5,701,145)	(256.52–285.31)	(8,005,261–9,688,169)	(301.53–364.46)	(0.1–0.36)	(−0.09–0.16)
Europe	5,170,116	755.85	5,599,167	722.77	−0.04	−0.03
	(4,961,553–5,350,086)	(729.75–777.58)	(5,324,503–5,862,790)	(691.59–754.69)	(−0.08–−0.01)	(−0.05–−0.01)
Iran (Islamic Republic of)	113,208	352.26	236,268	513.47	0.46	−0.05
	(104,437–121,563)	(325.48–378.53)	(214,190–262,927)	(465.78–568.24)	(0.31–0.62)	(−0.13–0.05)
**Iranian Provinces**						
Alborz	4403	452.40	10,023	613.21	0.36	−0.05
	(3767–5228)	(391.42–528.26)	(7675–12,932)	(478.7–780.5)	(0.04–0.8)	(−0.26–0.24)
Ardebil	1049	192.71	2270	309.56	0.61	−0.04
	(910–1212)	(168–221.27)	(1825–2718)	(253.1–366.99)	(0.26–1)	(−0.19–0.15)
Bushehr	1288	347.47	2888	495.29	0.43	−0.05
	(1106–1493)	(299.67–400.57)	(2326–3626)	(404.42–613.97)	(0.14–0.77)	(−0.21–0.15)
Chahar Mahaal and Bakhtiari	573	161.39	1222	230.73	0.43	−0.03
	(485–682)	(137.75–189.78)	(963–1533)	(183.95–287.9)	(0.11–0.82)	(−0.24–0.2)
East Azarbayejan	5198	293.14	10,997	459.69	0.57	−0.05
	(4365–5999)	(248.52–335.89)	(8671–14,249)	(362.88–584.26)	(0.19–1.03)	(−0.26–0.18)
Fars	7022	350.07	14,452	501.45	0.43	−0.06
	(6071–8062)	(303.58–401.68)	(11,453–18,156)	(401–622.17)	(0.12–0.82)	(−0.27–0.21)
Gilan	5409	397.57	9799	555.68	0.40	−0.06
	(4561–6326)	(336.67–462.97)	(7916–12,276)	(448.85–696.19)	(0.09–0.82)	(−0.27–0.21)
Golestan	1814	243.00	4213	397.36	0.64	−0.05
	(1514–2105)	(202.79–280.24)	(3363–5289)	(321.88–494.01)	(0.27–1.15)	(−0.22–0.14)
Hamadan	1951	239.62	3512	343.66	0.43	−0.04
	(1701–2254)	(210.63–275.53)	(2856–4359)	(281.23–422.25)	(0.14–0.76)	(−0.21–0.18)
Hormozgan	1162	223.07	2846	335.18	0.50	−0.03
	(936–1436)	(181.45–271.87)	(2108–3748)	(253.88–433.9)	(0.08–1.02)	(−0.22–0.23)
Ilam	510	226.09	1116	331.84	0.47	−0.05
	(431–601)	(193.41–263.98)	(872–1393)	(261.75–409.56)	(0.13–0.84)	(−0.24–0.16)
Isfahan	8258	367.13	16,190	515.36	0.40	−0.06
	(6974–9494)	(312.09–417.78)	(12,909–20,644)	(415.11–652.23)	(0.09–0.77)	(−0.25–0.18)
Kerman	2854	259.35	5976	364.52	0.41	−0.05
	(2450–3337)	(224.49–298.81)	(4659–7432)	(289.71–449.09)	(0.09–0.78)	(−0.23–0.22)
Kermanshah	2463	280.32	4703	396.14	0.41	−0.05
	(2149–2811)	(246.14–316.27)	(3736–5810)	(316.86–488.25)	(0.11–0.79)	(−0.24–0.22)
Khorasan-e-Razavi	5802	229.08	12,840	367.96	0.61	−0.05
	(4890–6625)	(194.63–260.77)	(10,145–16,262)	(294.97–464.88)	(0.24–1.04)	(−0.23–0.19)
Khuzestan	5729	338.93	12,474	518.62	0.53	−0.04
	(4933–6587)	(289.56–388.42)	(9826–15,844)	(414.3–646.81)	(0.15–0.96)	(−0.21–0.18)
Kohgiluyeh and Boyer-Ahmad	442	162.93	1010	270.33	0.66	−0.03
	(369–528)	(136.84–194.78)	(777–1264)	(213.62–336.65)	(0.25–1.21)	(−0.21–0.23)
Kurdistan	1304	205.36	2898	308.44	0.50	−0.04
	(1130–1495)	(179.19–234)	(2311–3593)	(247.09–381.3)	(0.17–0.9)	(−0.19–0.17)
Lorestan	1672	236.63	3539	358.84	0.52	−0.05
	(1400–2003)	(200.32–280.25)	(2798–4611)	(288.44–458.64)	(0.14–0.95)	(−0.25–0.18)
Markazi	1951	293.96	3540	410.20	0.40	−0.05
	(1674–2234)	(252.6–335.91)	(2887–4338)	(335.59–500.39)	(0.1–0.75)	(−0.25–0.16)
Mazandaran	6604	425.01	13,509	605.36	0.42	−0.06
	(5673–7520)	(369.31–479.13)	(10,772–16,752)	(485.7–748.87)	(0.12–0.78)	(−0.27–0.18)
North Khorasan	735	207.28	1491	333.20	0.61	−0.03
	(628–869)	(177–243.26)	(1166–1884)	(263.63–418.86)	(0.22–1.07)	(−0.22–0.19)
Qazvin	1605	313.40	3406	455.35	0.45	−0.06
	(1394–1852)	(274.23–359.33)	(2765–4158)	(373.95–556.56)	(0.16–0.82)	(−0.23–0.18)
Qom	1999	459.11	4331	629.97	0.37	−0.03
	(1710–2309)	(397.56–524.52)	(3418–5472)	(504.76–784.1)	(0.08–0.73)	(−0.21–0.18)
Semnan	1102	383.18	2051	489.56	0.28	−0.12
	(944–1266)	(330.87–439.48)	(1596–2544)	(382.12–610.01)	(0.01–0.63)	(−0.32–0.13)
Sistan and Baluchistan	901	122.83	2185	204.48	0.66	0.00
	(744–1107)	(103.39–148.34)	(1640–2801)	(157.61–257.46)	(0.25–1.19)	(−0.22–0.26)
South Khorasan	654	231.69	1545	352.66	0.52	−0.03
	(565–749)	(200.34–265.04)	(1227–1937)	(282.21–437.48)	(0.2–0.89)	(−0.2–0.17)
Tehran	33,095	593.06	69,620	825.15	0.39	−0.05
	(27,850–39,222)	(502.13–701.54)	(55,626–88,474)	(662.87–1044.04)	(0.06–0.82)	(−0.27–0.25)
West Azarbayejan	2910	225.54	6655	367.75	0.63	−0.04
	(2463–3430)	(192.73–262.87)	(5278–8309)	(296.28–454.89)	(0.25–1.09)	(−0.22–0.19)
Yazd	1950	446.34	3344	579.17	0.30	−0.08
	(1676–2260)	(384.54–515.51)	(2651–4178)	(465.84–717.38)	(0.03–0.68)	(−0.28–0.19)
Zanjan	800	182.25	1622	264.20	0.45	−0.04
	(684–942)	(156.78–212)	(1319–2022)	(217.23–324.99)	(0.16–0.85)	(−0.2–0.16)

**Table 4:** Comparison of DALY cases and rates per 100,000 of FBC based on provinces in 2010 and 2021.

Location	2010		2021		% Changes	
	DALY Number	DALY Rate per 100,000	DALY Number	DALY Rate per 100,000	2010–2021	2019–2021
	(95%UI)	(95%UI)	(95%UI)	(95%UI)	(95%UI)	(95%UI)
Global	15,925,184	456.88	20,254,802	455.56	0.00	0.00
	(15,230,377–16,586,720)	(436.88–475.96)	(18,963,376–21,574,429)	(426.64–485.3)	(−0.06–0.06)	(−0.06–0.06)
**Continents**						
Africa	1,514,823	511.67	2,474,148	580.09	0.13	0.02
	(1,367,445–1,690,636)	(464.47–568.56)	(2,137,127–2,839,076)	(507.7–662.36)	(0–0.3)	(−0.07–0.12)
America	2,920,320	548.61	3,467,032	516.39	−0.06	0.00
	(2,772,778–3,047,760)	(521.41–571.94)	(3,259,631–3,683,136)	(486.29–548.31)	(−0.1–−0.02)	(−0.03–0.02)
Asia	7,367,703	360.77	10,335,176	390.73	0.08	0.01
	(6,984,552–7,792,431)	(342.14–381.71)	(9,280,423–11,485,400)	(351.06–433.65)	(−0.03–0.2)	(−0.1–0.13)
Europe	4,086,140	615.63	3,939,488	526.84	−0.14	−0.04
	(3,875,839–4,273,871)	(589.92–642.21)	(3,657,964–4,200,047)	(493.64–560.95)	(−0.19–−0.1)	(−0.07–0)
Iran (Islamic Republic of)	92,343	280.98	152,966	327.45	0.17	−0.08
	(85,691–98,635)	(260.11–299.93)	(136,982–170,861)	(293.54–365.72)	(0.05–0.31)	(−0.16–0.01)
**Iranian Provinces**						
Alborz	3205	321.89	5995	363.85	0.13	−0.08
	(2694–3836)	(270.63–381.74)	(4417–7909)	(271.17–476.21)	(−0.16–0.53)	(−0.32–0.25)
Ardebil	866	155.58	1574	211.77	0.36	−0.07
	(740–1003)	(133.17–180.21)	(1241–1947)	(169.33–260.34)	(0.03–0.74)	(−0.24–0.14)
Bushehr	1150	301.69	1924	324.12	0.07	−0.08
	(972–1339)	(256.61–348.14)	(1528–2445)	(258.93–409.12)	(−0.15–0.39)	(−0.27–0.15)
Chahar Mahaal and Bakhtiari	428	117.38	678	125.51	0.07	−0.07
	(357–516)	(97.35–140.66)	(509–879)	(93.33–162.21)	(−0.23–0.43)	(−0.31–0.21)
East Azarbayejan	4919	274.20	7950	331.06	0.21	−0.09
	(4077–5681)	(227.33–316.76)	(6001–10,457)	(252.73–432.85)	(−0.11–0.62)	(−0.33–0.17)
Fars	6200	303.81	9728	334.06	0.10	−0.10
	(5254–7146)	(257.89–351.64)	(7419–12,611)	(258.41–429.34)	(−0.16–0.43)	(−0.32–0.22)
Gilan	4158	303.96	6026	340.55	0.12	−0.09
	(3463–4885)	(254.39–355.81)	(4577–7854)	(258.51–441.44)	(−0.16–0.48)	(−0.32–0.21)
Golestan	1809	235.26	3531	326.97	0.39	−0.09
	(1487–2096)	(193.7–274.76)	(2754–4497)	(255.94–418.04)	(0.04–0.9)	(−0.26–0.14)
Hamadan	1799	217.42	2558	247.89	0.14	−0.07
	(1559–2084)	(189.13–251.65)	(1990–3205)	(193.45–309.85)	(−0.13–0.44)	(−0.28–0.18)
Hormozgan	1207	223.63	2182	249.55	0.12	−0.08
	(959–1503)	(177.24–276.3)	(1551–2918)	(181.42–326.1)	(−0.24–0.56)	(−0.28–0.21)
Ilam	432	187.06	744	217.05	0.16	−0.09
	(361–508)	(158.07–219.71)	(566–928)	(165.4–271.59)	(−0.12–0.5)	(−0.3–0.15)
Isfahan	6436	281.84	10,144	319.01	0.13	−0.09
	(5339–7479)	(234.2–326.16)	(7762–13,065)	(244.72–408.5)	(−0.16–0.47)	(−0.31–0.19)
Kerman	2716	240.94	4504	269.36	0.12	−0.08
	(2306–3218)	(205.72–282.85)	(3423–5780)	(208.18–343.95)	(−0.17–0.45)	(−0.29–0.22)
Kermanshah	2362	263.12	3406	284.04	0.08	−0.08
	(2053–2724)	(228.47–301.9)	(2669–4389)	(223.81–365.93)	(−0.18–0.41)	(−0.3–0.24)
Khorasan-e-Razavi	6102	237.10	9954	280.74	0.18	−0.10
	(5135–6982)	(200.57–272.92)	(7625–12,871)	(215.23–362.82)	(−0.12–0.54)	(−0.3–0.16)
Khuzestan	5322	305.09	9066	369.78	0.21	−0.08
	(4528–6133)	(259.14–353.19)	(6992–11,656)	(288.77–473.07)	(−0.11–0.59)	(−0.26–0.16)
Kohgiluyeh and Boyer-Ahmad	352	125.54	624	161.77	0.29	−0.07
	(287–427)	(101.41–151.98)	(462–809)	(120.41–208.79)	(−0.09–0.76)	(−0.28–0.23)
Kurdistan	1252	193.05	2039	214.00	0.11	−0.08
	(1068–1442)	(165.68–220.79)	(1597–2561)	(167.95–270.17)	(−0.15–0.43)	(−0.25–0.15)
Lorestan	1398	192.75	2226	222.38	0.15	−0.08
	(1167–1668)	(161.75–227.38)	(1711–2880)	(171.09–286.76)	(−0.14–0.54)	(−0.31–0.19)
Markazi	1701	253.89	2367	271.32	0.07	−0.09
	(1441–1978)	(214.66–295.53)	(1878–2964)	(214.73–339.7)	(−0.18–0.4)	(−0.29–0.15)
Mazandaran	4808	304.25	8096	359.06	0.18	−0.09
	(4072–5467)	(257.8–347)	(6384–10,267)	(283.28–453.4)	(−0.08–0.52)	(−0.31–0.19)
North Khorasan	738	203.22	1201	263.53	0.30	−0.07
	(616–895)	(169.98–245.55)	(905–1580)	(199.93–346.77)	(−0.07–0.72)	(−0.28–0.18)
Qazvin	1403	268.45	2305	302.28	0.13	−0.09
	(1219–1620)	(232.7–311.39)	(1846–2893)	(241.83–383.18)	(−0.12–0.44)	(−0.28–0.17)
Qom	1776	404.85	2828	408.07	0.01	−0.06
	(1506–2076)	(348.88–468.66)	(2195–3643)	(321.43–525.55)	(−0.22–0.3)	(−0.25–0.18)
Semnan	940	322.29	1353	318.11	−0.01	−0.16
	(806–1094)	(277.18–374.91)	(976–1740)	(229.4–409.85)	(−0.26–0.31)	(−0.39–0.14)
Sistan and Baluchistan	1209	156.56	2329	208.05	0.33	−0.06
	(983–1472)	(127.15–187.41)	(1720–3104)	(154.77–275.96)	(−0.06–0.84)	(−0.3–0.25)
South Khorasan	620	218.00	1144	257.80	0.18	−0.07
	(530–722)	(186.78–254.55)	(901–1450)	(204.61–326.57)	(−0.1–0.51)	(−0.25–0.16)
Tehran	21,950	386.37	38,343	450.03	0.16	−0.07
	(18,138–26,780)	(318.67–469.14)	(29,599–48,898)	(349.56–572.58)	(−0.16–0.58)	(−0.31–0.3)
West Azarbayejan	2743	208.41	4951	270.72	0.30	−0.09
	(2305–3301)	(176.08–247.79)	(3790–6371)	(209.48–345.23)	(−0.04–0.75)	(−0.28–0.19)
Yazd	1669	377.12	2145	364.19	−0.03	−0.12
	(1421–1931)	(321.08–432.22)	(1639–2787)	(279.27–469.93)	(−0.26–0.28)	(−0.34–0.2)
Zanjan	672	150.08	1049	168.11	0.12	−0.08
	(565–799)	(126.66–176.71)	(833–1333)	(134.18–212.58)	(−0.16–0.47)	(−0.25–0.15)

## Data Availability

The data supporting the findings of this study are openly available. The definitions for the terms and variables used can be found at the Global Health Data Exchange (GHDx): https://www.healthdata.org/terms-defined and https://www.healthdata.org/gbd/ on 17 July 2025.
